# Prediction of the tetramer protein complex interaction based on CNN and SVM

**DOI:** 10.3389/fgene.2023.1076904

**Published:** 2023-01-26

**Authors:** Yanfen Lyu, Ruonan He, Jingjing Hu, Chunxia Wang, Xinqi Gong

**Affiliations:** ^1^ Department of Mathematics and PhysicsScience and Engineering, Hebei University of Engineering, Handan, China; ^2^ School of Information, Renmin University of China, Beijing, China; ^3^ School of Landscape and Ecological Engineering, Hebei University of Engineering, Handan, China; ^4^ Mathematical Intelligence Application Lab, Institute for Mathematical Sciences, School of Math, Renmin University of China, Beijing, China; ^5^ Beijing Academy of Artificial Intelligence, Beijing, China

**Keywords:** tetramer protein complex interaction, feature map, CNN, SVM ensemble method, under-sampling

## Abstract

Protein-protein interactions play an important role in life activities. The study of protein-protein interactions helps to better understand the mechanism of protein complex interaction, which is crucial for drug design, protein function annotation and three-dimensional structure prediction of protein complexes. In this paper, we study the tetramer protein complex interaction. The research has two parts: The first part is to predict the interaction between chains of the tetramer protein complex. In this part, we proposed a feature map to represent a sample generated by two chains of the tetramer protein complex, and constructed a Convolutional Neural Network (CNN) model to predict the interaction between chains of the tetramer protein complex. The AUC value of testing set is 0.6263, which indicates that our model can be used to predict the interaction between chains of the tetramer protein complex. The second part is to predict the tetramer protein complex interface residue pairs. In this part, we proposed a Support Vector Machine (SVM) ensemble method based on under-sampling and ensemble method to predict the tetramer protein complex interface residue pairs. In the top 10 predictions, when at least one protein-protein interaction interface is correctly predicted, the accuracy of our method is 82.14%. The result shows that our method is effective for the prediction of the tetramer protein complex interface residue pairs.

## 1 Introduction

Protein-protein interactions are significant in various biological activities and processes, such as signal transmission, gene expression and transcriptional regulation (Levy and Pereira-Leal, 2008; Malta et al., 2018; Li et al., 2019; Lyu et al., 2020; Zhao et al., 2022). The interactions between proteins in the body can form dimer protein complexes, trimer protein complexes, tetramer protein complexes and higher polymers. The more monomers in a polymer, the more complex its internal interactions become. Therefore, studying protein-protein interactions contributes to a better understanding of the formation mechanism of multibody protein complexes (Gao and Skolnick, 2012; Sun et al., 2020a). Under certain conditions, some protein-protein interaction interface residue pairs are functional sites of protein complexes and are associated with certain diseases (Oganesyan et al., 2004; McKinstry et al., 2009; Vidal et al., 2011; Li et al., 2021a; Baek et al., 2021). If the interface residue pairs of protein-protein interaction can be provided, it will be great helpful for the multibody protein complex structural design, protein complex function prediction and drug design (Yang et al., 2015; Zhang et al., 2017; Zhang et al., 2017).

With the development of technology, some experimental methods can be used to study the interactions of multibody protein complexes, such as X-ray crystallography, Cryogenic electron microssopy (Cryo-EM) and Nucleic Magnetic Resonance (NMR) (Drennan et al., 1994; Sun et al., 2020b). These experimental methods have made great contributions to our understanding of the protein complex interaction mechanism. However, due to experimental conditions or technical limitations, it is impossible to use experimental methods to study all protein complex interactions. For example, X-ray crystallography method can only be used to study some protein complexes that can form stable crystals. When NMR method is used to study protein complex interactions, the size of protein complex is limited. However we have accumulated a number of protein complex data through these experimental methods, which provide the data basis for computing methods to study protein complex interactions.

At present, researchers have developed several calculation methods to predict protein complex interactions, such as Wang et al. proposed to use different machine learning methods to predict different types of protein-protein interaction interface residue pairs (Wang et al., 2017). Ovchinnikov proposed a method based on evolutionary information to predict protein-protein interaction interface residue pairs (Ovchinnikov et al., 2014). Du et al. used depth learning technology (stacked automatic encoder) to build a deep neural network model to tackle the residue-residue contact prediction problem (Du et al., 2016). Liu et al. used an attention mechanism enhanced Long Short Term Memory (LSTM) model to predict dimer protein complex interface residue pairs (Liu and Gong, 2019). Martin et al. predicted residue contact in protein-protein interaction by message passing (Weigt et al., 2009). We also developed a two-layer support vector machine ensemble classifier to predict trimer protein complex interface residue pairs (Lyu and Gong, 2020). There are many other methods, see references (Kamisetty et al., 2013; Fu et al., 2014; Michel et al., 2014; He et al., 2017; Li et al., 2019; Zhang et al., 2020; Li et al., 2021a; Li et al., 2021b; Humphreys et al., 2021; Jumper et al., 2021; Mylonas et al., 2021; Knutson et al., 2022). These methods have achieved good results in the study of protein complex interaction, but most of them focus on the study of dimer and trimer protein complex interaction, and few on the study tetramer protein complex interaction. Sun et al. developed a deep network based on LSTM network with a graph to predict the tetramer protein complex interface residue pairs, but their method did not consider whether the chains of the tetramer protein complex interact with each other (Sun and Gong, 2020). Predicting protein-protein interactions and non-interactions is very important for the study of multibody protein interactions (Humphreys et al., 2021; Zhao et al., 2022). Thus, new methods are needed for studying tetramer protein interaction.

To further improve above mentioned defections, we have done two parts of work on the study of tetramer protein complex interaction. The first part is to predict the interaction between chains of the tetramer protein complex. The second part is to predict the tetramer protein complex interface residue pairs, that is, assuming that the interaction between two chains of the tetramer protein complex is known, we predict the interface residue pairs formed by the interaction.

In first part, according to the five geometric properties of residue, the protein sequence was mapped into five number sequences. Based on these number sequences, we defined the position change sequence and geometric feature change sequences of the same type of amino acids. Then combined with four mathematical statistics, we extracted a 20 × 24 feature map to represent a sample generated by two chains of the tetramer protein complex. Finally, we constructed a CNN model based on PyTorch framework to predict the interaction between chains of the tetramer protein complex.

In second part, the influence of surrounding amino acids (residues) on the central amino acid (the central residue) is fully considered in feature extraction. We defined the Amino Acid *k*-Average Cumulation Factor, and combined the Amino Acid *k*-Interval Product Factor to extract features based on protein sequence. We also defined the Residue *k*-Interval Product Factor, Residue *k*-Average Cumulation Factor and weight factor to extract features based on protein three-dimensional structure. Finally, we proposed a SVM ensemble method to predict the tetramer protein complex interface residue pairs.

## 2 Materials and methods

### 2.1 Dataset

In this paper, we collect 111 tetramer protein complexes from the Protein Data Bank according to the following three requirements: the number of chains in the protein complex is 4, the number of amino acids in each chain is between 20 and 500, its crystal structure is obtained by X-ray experimental method. The PDB ID of these 111 protein tetramers is shown in Supplementary Table S1. If the contact area between any two atoms from two residues of two chains is bigger than zero, we call these two residues an interface residue pair (Lyu and Gong, 2020). The contact area between two atoms is calculated by Qcontacts software. If there is at least one interface residue pair between two chains of the tetramer protein complex, we call the two chains interacting, otherwise the two chains are not interacting.

### 2.2 Construct feature map and CNN model to predict the interaction between chains of the tetramer protein complex

#### 2.2.1 Construct feature map

For protein sequence P with length *L*, see formula 1. In protein P three-dimensional structure, different amino acids have different geometric properties. These geometric properties, such as Accessible Surface Area (ASA), Relative solvent Accessible Surface Area (RASA), Exterior Contact Area (ECA), Interior Contact Area (ICA), and Exterior Void Area (EVA), play important roles in multibody protein complex interactions (Wang et al., 2017; Yang and Gong, 2018; Liu and Gong, 2019; Zhao and Gong, 2019; Lyu and Gong, 2020; Sun and Gong, 2020). In this paper, we consider using the above five geometric properties to predict the tetramer protein complex interaction. References (Liu and Gong, 2019; Lyu and Gong, 2020) and (Zhao and Gong, 2019) introduce the five geometric properties and their computing tools in detail.

$$P=P_{1}P_{2}\cdots P_{L}$$ (1)

Where $P_{j}\in \Omega ,\;\Omega =\left\{A,\;C,\;D,\;E,\;F,\;G,\;H,\;I,\;K,\;L,\;M,\;N,\;P,\;Q,\;R,\;S,\;T,\;V,\;W,\;Y\right\},\;A,C,\ldots ,Y$ is the abbreviation of amino acid name.

According to the 5 geometric properties of each amino acid, the protein sequence P is mapped into 5 number sequences, see formula 2. We used P^1^, P^2^, P^3^, P^4^ and P^5^ to represent the 5 number sequences. These 5 number sequences are the ASA number sequence, RASA number sequence, ECA number sequence, ICA number sequence and EVA number sequence.

$$P^{i}=\varphi_{1}^{i}\varphi_{2}^{i}∙∙∙\varphi_{L}^{i}\;\left(i=1,2,3,4,5\right)$$ (2)

Where $\varphi_{1}^{1}$ is the ASA value of P_1_ in formula 1, $\varphi_{2}^{1}$ is the ASA value of P_2_ in formula 1, and so on. $\varphi_{1}^{2}$ is the RASA value of P_1_ in formula 1, $\varphi_{2}^{2}$ is the RASA value of P_2_ in formula 1, and so on. $\varphi_{1}^{3}$ is the ECA value of P_1_ in formula 1, $\varphi_{2}^{3}$ is the ECA value of P_2_ in formula 1, and so on. $\varphi_{1}^{4}$ is the ICA value of P_1_ in formula 1, $\varphi_{2}^{4}$ is the ICA value of P_2_ in formula 1, and so on. $\varphi_{1}^{5}$ is the EVA value of P_1_ in formula 1, $\varphi_{2}^{5}$ is the EVA value of P_2_ in formula 1, and so on.

For any amino acid x∈Ω , suppose that x occurs *n* times in protein sequence P, the occurrence positions from left to right are $\alpha_{1}{,\alpha }_{2},∙∙∙\alpha_{n}$ respectively, and the corresponding values in the number sequence are $\beta_{1}^{i},\beta_{2}^{i},∙∙∙,\beta_{n}^{i}$ ( i=1,2,3,4,5 ) respectively.

We define the same type of amino acid position change sequence $f_{x}\left(\tau \right)$ , as following:

$$f_{x}\left(\tau \right)=\left\{\;\begin{array}{l} {\;\alpha }_{\tau +1}-\alpha_{\tau }\;1\leq \tau \leq n-1\;\left(n>1\right)\\ \alpha_{1}-\frac{\overset{L}{\underset{j=1}{\sum }}j}{L}\;\left(n=1\right)\\ 0\;\left(n=0\right) \end{array}\right.$$ (3)

We define the same type of amino acid geometry feature change sequence $f_{x}^{i}\left(\tau \right)$ , as following:

$$f_{x}^{i}\left(\tau \right)=\left\{\;\begin{array}{l} \;\beta_{\tau +1}^{i}-\beta_{\tau }^{i}\;1\leq \tau \leq n-1\;\left(n>1\right)\\ \beta_{1}^{i}-\frac{\overset{L}{\underset{\tau =1}{\sum }}\beta_{\tau }^{i}}{L}\;\left(n=1\right)\\ \;0\;\left(n=0\right) \end{array}\;\right.\;\left(i=1,2,3,4,5\right)$$ (4)

The monomer protein can be represent by the same type of amino acid position change sequence $f_{x}\left(\tau \right)$ and the same type of amino acid geometry feature change sequences $f_{x}^{i}\left(\tau \right)$ . This representation method based on amino acid position and geometric features change sequences preserves the important information of protein sequence and three-dimensional structure, so it is feasible to apply it to protein complex interaction prediction.

Based on the same type of amino acid position change sequence $f_{x}\left(\tau \right)$ and the same type of amino acid geometry feature change sequences $f_{x}^{i}\left(\tau \right)$ , we extract 24 features.

Firstly, we extract four mathematical statistics from the same type of amino acid position change sequence $f_{x}\left(\tau \right)$ as follows:

(1). The frequency of amino acid *x*, denoted as $F_{x}$ , see formula 5. $\left|f_{x}\left(\tau \right)\right|$ represents the length of the same type of amino acid position change sequence $f_{x}\left(\tau \right)$ .

$$F_{x}=\frac{\left|f_{x}\left(\tau \right)\right|+1}{L}$$ (5)

(2). The arithmetic mean of the same type of amino acid *x* position change sequence $f_{x}\left(\tau \right)$ , denoted as $A_{x}$ , see formula 6.

$$A_{x}=\left\{\begin{array}{l} f_{x}\left(1\right)\;\left(n=1\right)\\ \frac{\sum_{\tau =1}^{n-1}f_{x}\left(\tau \right)}{\left|f_{x}\left(\tau \right)\right|}\;\left(n>1\right)\\ 0\;\left(n=0\right) \end{array}\right.$$ (6)

(3). The minimum of the same type of amino acid position *x* change sequence $f_{x}\left(\tau \right)$ , denoted as $B_{x}$ , see formula 7.

$$B_{x}=\left\{\begin{array}{l} \;\min\left(f_{x}\left(\tau \right)\right)\;\left(n\geq 1\right)\\ \;0\;\left(n=0\right) \end{array}\right.$$ (7)

(4). The maximum of the same type of amino acid *x* position change sequence $f_{x}\left(\tau \right)$ , denoted as $M_{x}$ , see formula 8.

$$M_{x}=\left\{\begin{array}{l} \;\max\left(f_{x}\left(\tau \right)\right)\;\left(n\geq 1\right)\\ 0\;\left(n=0\right) \end{array}\right.$$ (8)

Secondly, we extract four mathematical statistics from the same type of amino acid geometry feature change sequence $f_{x}^{i}\left(\tau \right)$ , as follows:

(1). The arithmetic mean of the same type of amino acid *x* geometry feature change sequence $f_{x}^{i}\left(\tau \right)$ , denoted as $A_{x}^{i}$ , see formula 9.

$$A_{x}^{i}=\left\{\begin{array}{l} \;\frac{\underset{\tau }{\sum }f_{x}^{i}\left(\tau \right)}{\left|f_{x}^{i}\left(\tau \right)\right|}\;\left(n\geq 1\right)\\ \;0\;\left(n=0\right) \end{array}\right.\;\left(i=1,2,3,4,5\right)$$ (9)

(2). The minimum of the same type of amino acid *x* geometry feature change sequence $f_{x}^{i}\left(\tau \right)$ , denoted as $B_{x}^{i}$ , see formula 10.

$$B_{x}^{i}=\left\{\begin{array}{l} \;\min\left(f_{x}^{i}\left(\tau \right)\right)\;\left(n\geq 1\right)\\ \;0\;\left(n=0\right) \end{array}\right.\;\left(i=1,2,3,4,5\right)$$ (10)

(3). The maximum of the same type of amino acid *x* geometry features change sequence $f_{x}^{i}\left(\tau \right)$ , denoted as $M_{x}^{i}$ , see formula 11.

$$M_{x}^{i}=\left\{\begin{array}{l} \;\max\left(f_{x}^{i}\left(\tau \right)\right)\;\left(n\geq 1\right)\\ \;0\;\left(n=0\right) \end{array}\right.\;\left(i=1,2,3,4,5\right)$$ (11)

(4). The ratio of the arithmetic mean of the same type of amino acid *x* geometry feature change sequence to the arithmetic mean of the same type of amino acid *x* position change sequence, denoted as $R_{x}^{i}$ , see formula 12.

$$R_{x}^{i}=\frac{A_{x}^{i}}{A_{x}}\;\left(i=1,2,3,4,5\right)$$ (12)

According to the above statistics, we obtain 4+4×5 = 24 features to characterize each type of amino acid. The monomer protein is composed of 20 types of amino acids. So we use a 20 × 24 dimension matrix Q to represent each monomer protein, as shown in formula 13.

$$Q=\left[\begin{array}{ccc} q_{1,1} & \cdots & q_{1,24}\\ \vdots & \ddots & \vdots \\ q_{20,1} & \cdots & q_{20,24} \end{array}\right]$$ (13)

The line represents the number of amino acid types, and the column shows 24 features of each type of amino acid.

In order to better understand 24 features calculation process, we give an example as follows:

For protein sequence P = ACAGAHHAALKAYAW, we calculate 24 features of the A amino acid. According to the definition of amino acid position change sequence, we can get $f_{A}$ = 2 2 3 1 3 2. Then, we use Qcontacts software to calculate the ASA value of each amino acid on protein sequence P, so as to obtain the ASA number sequence $P^{1}$ = 6 4 7 5 7 8 2 8 9 3 7 11 10 14 15. According to the definition of amino acid geometry feature change sequence, we can get $f_{A}^{1}$ = 1 0 1 1 2 3.

Applying formula 5, formula 6, formula 7, formula 8, formula 9, formula 10, formula 11, formula 12):

$$F_{A}=\frac{7}{15},\;A_{A}=\frac{13}{7},\;B_{A}=1,\;M_{A}=3$$

$$A_{A}^{1}=\frac{8}{7},\;B_{A}^{1}=0,\;M_{A}^{1}=3,\;R_{A}^{1}=\frac{8}{3}$$

The calculation process of other four geometry feature change sequences statistics is the same as that of ASA feature change sequencesstatistics. So we can obtain 4+4×5 = 24 features to characterize A amino acid.

A tetramer protein complex is composed of four chains, and any two chains can generate a sample, so a tetramer protein complex can generate six samples. We use a 20 × 24 dimension matrix S to represent a sample, where the matrix S is generated by the absolute value of the difference between the corresponding feature values of two matrices generated by two chains. For example 1REW_ABCD, 1REW is the tetramer protein complex PDB ID. A, B, C and D are the names of four chains. We use $Q_{A}$ , $Q_{B}$ , $Q_{C}$ and $Q_{D}$ to represent 20 × 24 dimension matrixes generated by four chains respectively. A total of six samples are generated from 1REW protein tetramer, as follows:

Sample 1 generated by A chain and B chain: $S_{1}={\left(\left|a_{uv}-b_{uv}\right|\right)}_{20\times 24}$

Sample 2 generated by A chain and C chain: $S_{2}={\left(\left|a_{uv}-c_{uv}\right|\right)}_{20\times 24}$

Sample 3 generated by A chain and D chain: $S_{3}={\left(\left|a_{uv}-d_{uv}\right|\right)}_{20\times 24}$

Sample 4 generated by B chain and C chain: $S_{4}={\left(\left|b_{uv}-c_{uv}\right|\right)}_{20\times 24}$

Sample 5 generated by B chain and D chain: $S_{5}={\left(\left|b_{uv}-d_{uv}\right|\right)}_{20\times 24}$

Sample 6 generated by C chain and D chain: $S_{6}={\left(\left|c_{uv}-d_{uv}\right|\right)}_{20\times 24}$

Where $Q_{A}={\left(a_{uv}\right)}_{20\times 24},\;Q_{B}={\left(b_{uv}\right)}_{20\times 24},\;Q_{C}={\left(c_{uv}\right)}_{20\times 24},\;Q_{D}={\left(d_{uv}\right)}_{20\times 24}$ .

We use $Y_{r}$ to denote the sample label, $Y_{r}=1$ denotes that there is interaction between two chains and $Y_{r}=0$ denotes that there is no interaction between two chains.

**FIGURE 1:**
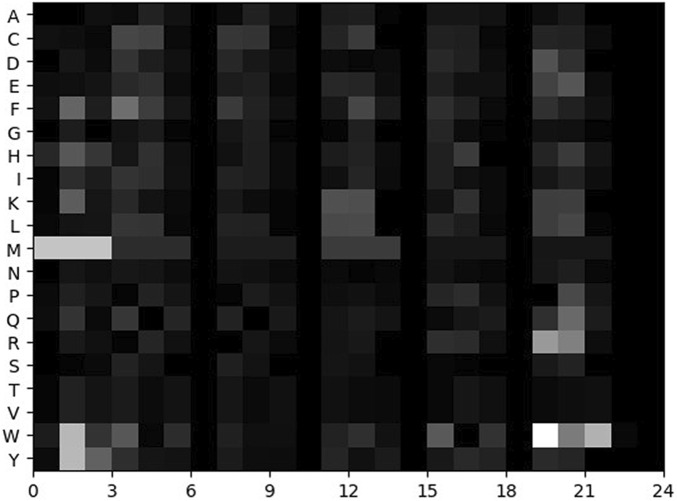
Feature map of a sample.

The matrix S of each sample is standardized by formula 14. The normalized matrix can be regarded as a greyscale image. The larger the value, the brighter the pixel. The smaller the value, the darker the pixel. The grayscale image is called the feature map, as shown in Figure 1.

$$x^{\prime }=\frac{x-mean}{\sigma }$$ (14)

#### 2.2.2 Construct a convolutional neural network (CNN) model

**FIGURE 2:**
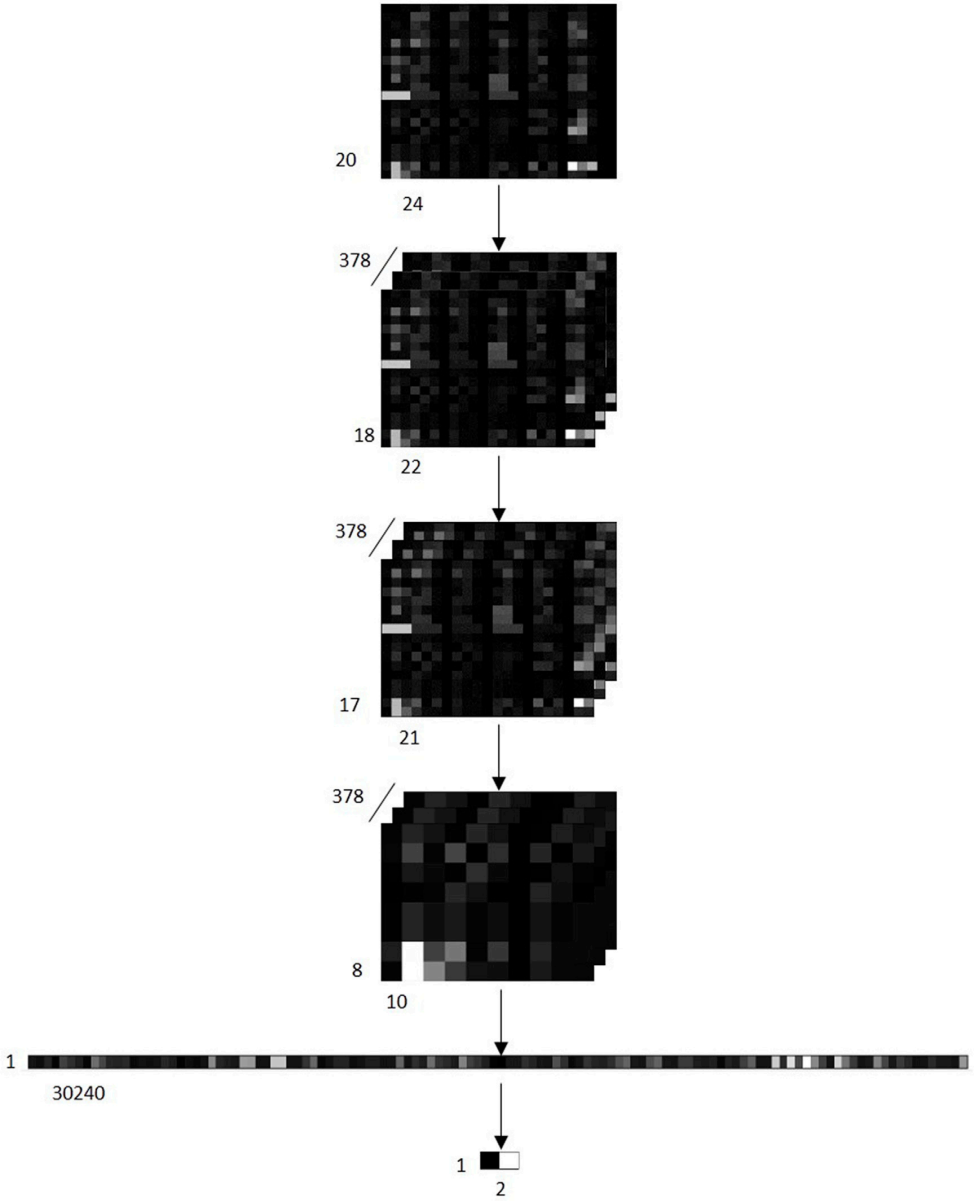
Schematic diagram of various transformations that occur after feature maps of samples are input into the CNN model. Input 378 feature maps into the CNN model. The first convolution layer generates 378 [18 × 22] matrixes. The second convolution layer converts the 378 [18 × 22] matrixes into 378 [17 × 21] matrixes. Next, it is converted into 378 [8 × 10] matrixes through the maximum pooling layer, and then expand the matrix into a [1 × 30240] Vector. Finally, a [1 × 2] vector is output through a full connection layer, where 0 represents that the sample is predicted to be a negative class, and 1 represents that the sample is predicted to be a positive class.

Convolutional Neural Network (CNN) is a kind of feedforward neural network with deep structure. The CNN model we created is based on PyTorch framework, which consists of 2 sets of convolution layer, a pooling layer and a full connected layer. In first convolution layer, we select 3 × 3 kernels slide over the input feature maps performing convolution operation (step size is 1), and process with the Rectified Linear Unit activation function. In second convolution layer, we use 2 × 2 kernels to perform convolution operation over feature maps (step size is 1), and also process with Rectified Linear Unit activation function. In the pooling layer, we collect the maximum values in every 2 × 2 patch of feature maps through a sliding window to form a more robust pooled feature maps. Then flatten it into a vector and output the results through a fully connected layer. Figure 2 shows the various transformations that occur after feature maps are input into the CNN model.

**FIGURE 3:**
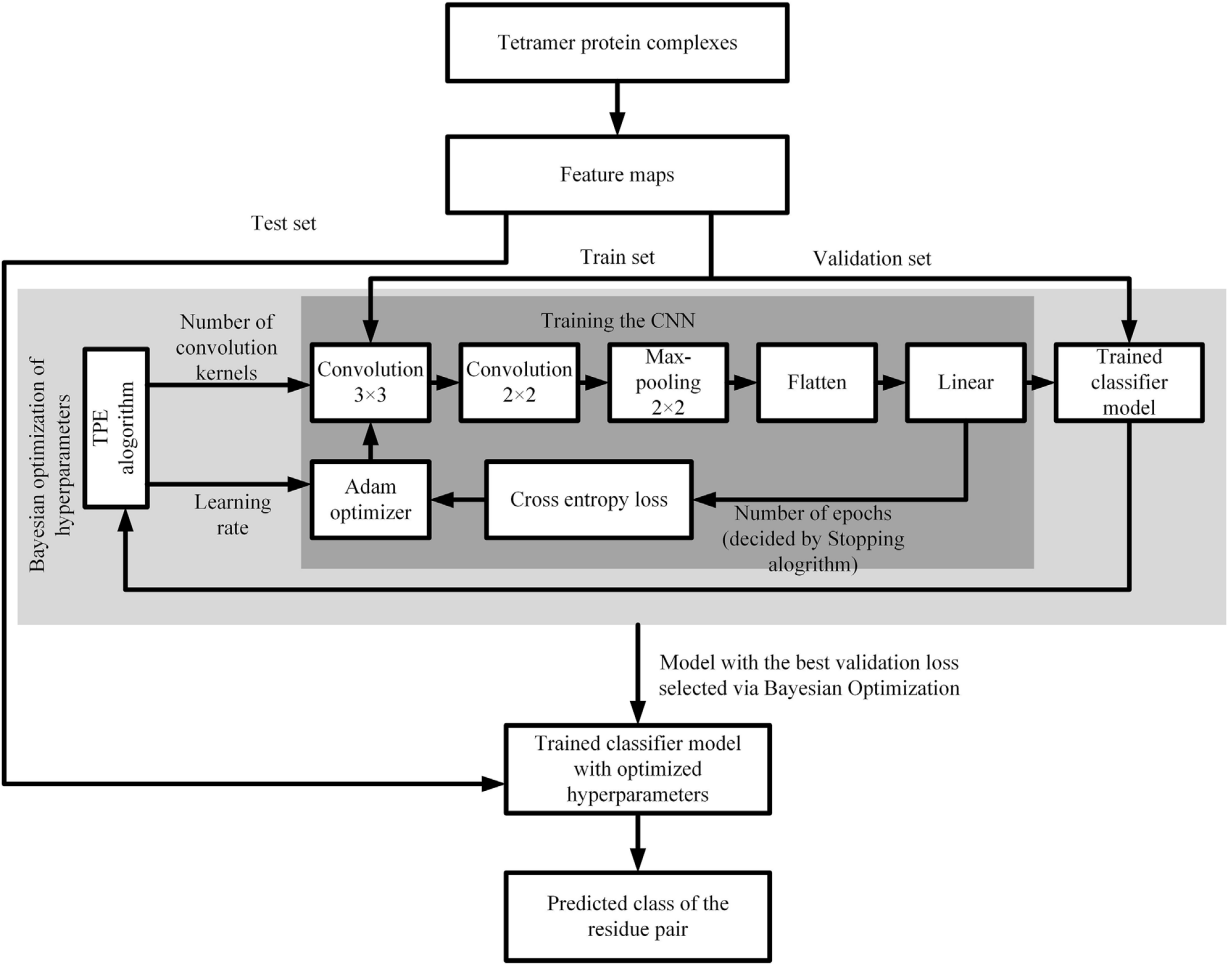
Flow chart of the CNN model.

The CNN model contains many hyper parameters that have different effects on its overall performance (Wardah et al., 2020). In this paper, we use bayesian optimization to select model hyper parameters. The batch size is set to 128, epoch is set to 100, the learning rate value is set between 0.00001 and 0.001, and loss function is cross entropy loss function. Using adam optimization algorithm to adjust the internal weight of the network. The flow chart of CNN model is shown in Figure 3.

### 2.3 Construct SVM ensemble method to predict the tetramer protein complex interface residue pairs

#### 2.3.1 Feature extraction

In this paper, for a given amino acid (we call it the central amino acid, whle the residue corresponding to the central amino acid in protein three-dimensional structure is called the central residue), we consider the influence of surrounding amino acids (residues) on the central amino acid (the central residue). Firstly, we consider the influence of each surrounding amino acid (residue) on the central amino acid (residue). Secondly, we take a certain amount of amino acids (residues) as a whole, and consider the influence of this whole on the central amino acid (the central residue) and the influence of each residue in the whole on the central residue.

##### 2.3.1.1 Sequence feature extraction

The physicochemical properties of different types of amino acids are different, and these physicochemical properties play important roles in protein-protein interactions. In this paper, we consider hydrophobicity, polarizability, polarity, secondary structure, and codon diversity of the amino acid, and values of these five physicochemical properties of each amino acid are shown in Supplementary Table S2 (Tanford, 1962; Grantham, 1974; Charton and Charton, 1982; Kyte and Doolittle, 1982; Lyu and Gong, 2020). For protein sequence P with length *L*, see formula 1. According to the five physicochemical properties of each amino acid, we map the protein sequence P to 5 number sequences, as shown in formula 15.

$$p^{i}=\Phi_{1}^{i}\Phi_{2}^{i}\cdots \Phi_{L}^{i}\;\left(i=1,2,3,4,5\right)$$ (15)

In protein-protein interactions, the individual behavior of the central amino acid is affected by the neighboring amino acids in the protein sequence. In our previous work, we defined the Amino Acid *k*-Interval Product Factor (AAIPF(*k*)^*i*^) to describe the influence of neighboring amino acids on the central amino acid (Lyu and Gong, 2020), see formula 16-18. Similarly, in protein-protein interactions, a certain number of amino acids around the central amino acid have an overall effect on the central amino acid. We define the Amino Acid *k* Average Cumulative Factor (AAACF(*k*)^*i*^) to describe the overall effect.

The Amino Acid *k* Average Cumulative Factor (AAACF(*k*)^*i*^) is defined as follows: for the central amino acid P_*j*_, in the number sequence, divide the sum of the value of the central amino acid position and its forward *k* positions and backward *k* positions by 2*k*+1, as shown in Formula 19.

$$AAIPF{\left(k\right)}^{i}=\left\{\begin{array}{l} AAFIPF{\left(k\right)}^{i}\\ AABIPF{\left(k\right)}^{i} \end{array}\right.$$ (16)

$$AAFIPF{\left(k\right)}^{i}=\frac{\Phi_{j}^{i}\times \Phi_{j-k}^{i}}{k}$$ (17)

$$AABIPF{\left(k\right)}^{i}=\frac{\Phi_{j}^{i}\times \Phi_{j+k}^{i}}{k}$$ (18)

$${AAACF\left(k\right)}^{i}=\frac{1}{2k+1}\sum_{\sigma =j-k}^{j+k}\Phi_{\sigma }^{i}$$ (19)

In the previous study (Afreixo et al., 2009; Lyu and Gong, 2020), the protein sequence P was regarded as a cycle alphabet sequence with head-to-tail connections to explore the individual behavior of each amino acid. Good results have been obtained by taking this strategy, so we also use this strategy in this paper. Considering the dimension of descriptors and using the experience of previous works (Wang and Brown, 2006; Wang et al., 2010; Lyu and Gong, 2020), we only consider the influence of before 10 amino acids and after 10 amino acids of the central amino acid. So we extract AAIPF(1)^*i*^, AAIPF(2)^*i*^, AAIPF(3)^*i*^, AAIPF(4)^*i*^, AAIPF(5)^*i*^, AAIPF(6)^*i*^, AAIPF(7)^*i*^, AAIPF(8)^*i*^, AAIPF(9)^*i*^ and AAIPF(10)^*i*^ to describe the effect of each amino acid on the central amino acid. We extract AAACF(1)^*i*^, AAACF(2)^*i*^, AAACF(3)^*i*^, AAACF(4)^*i*^, AAACF(5)^*i*^, AAACF(6)^*i*^, AAACF(7)^*i*^, AAACF(8)^*i*^, AAACF(9)^*i*^ and AAACF(10)^*i*^ to describe the effect of the whole formed by a certain number (3,5,7,…,21) of amino acids on the central amino acid. We also use the five physicochemical characteristics of the central amino acid as features to describe the amino acid. Thus we can use 5×(20+10)+5 = 155 features to describe each amino acid.

##### 2.3.1.2 Structure feature extraction

In several previous research studies (Yang and Gong, 2018; Liu and Gong, 2019; Zhao and Gong, 2019; Lyu and Gong, 2020; Sun and Gong, 2020), it has been found that the five geometric properties (ASA, RASA, ECA, ICA, and EVA) can be used to distinguish interface residues and non-interface residues. According to the five geometric properties of the residue, we map the protein P to 5 number sequences, as shown in formula 2.

For a given central residue, we calculate the Euclidean distance between each residue and the given central residue according to the three-dimensional coordinates of the $C_{\alpha }$ in the monomer protein PDB file and perform ascending sort. We use $\lambda_{1},\lambda_{2},\ldots ,\lambda_{L-1}$ to express the corresponding position of amino acids on the protein sequence P, and we use $d_{1},d_{2},\ldots ,d_{L-1}$ to express the sorted Euclidean distance.

In protein-protein interactions, the individual behavior of the central residue is affected by neighboring residues in the protein three-dimensional structure, we define Residue *k*-Interval Product Factor (RIPF(*k*)) to describe the effect. The RIPF(*k*) is defined as follows: on the monomer protein three-dimensional structure, for a given central residue P_*j*_, multiply the geometric value of the *k*th residue closest to the central residue by the geometric value of the central residue, and divide the product by *k* (see formula 20). When we regard the central residue and some residues closest the central residue as a whole, we define Residue *k*-Average Cumulative Factor (RACF(*k*)) and weight factor $\rho_{\xi }^{i}$ to describe the influence of the whole on the central residue and the influence of each residue in the whole on the central residue (see formula 21-22).

$${RIPF\left(k\right)}_{j}^{i}=\frac{\varphi_{j}^{i}\times \varphi_{\lambda_{k}}^{i}}{k}$$ (20)

Where $\lambda_{k}$ represents the position of the *k*-th residue closest to the central residue in the monomer protein three-dimensional structure.

$${RACF\left(k\right)}^{i}=\frac{\varphi_{j}^{i}+\sum_{ii=1}^{k}\varphi_{\lambda_{ii}}^{i}}{k+1}$$ (21)

$$\rho_{\xi }^{i}\left(k\right)=\omega_{\xi }\times \varphi_{\lambda_{\xi }}^{i}$$ (22)

$$\omega_{\xi }\left(k\right)=e^{-\frac{d_{\xi }^{2}\times \left(k+1\right)}{\sum_{\xi =1}^{k+1}d_{\xi }^{2}}}$$ (23)

Where (*k*+1) is the number of residues in the whole. $\omega_{\xi }$ is the weight of the ξ -th residue closest to the central residue.

In the protein three-dimensional structure, for the central residue, we consider the influence of 20 residues closest to the central residue. We extract ${RIPF\left(l\right)}_{j}^{i}\;\left(l=1,2,\ldots ,20\right)$ to represent the effect of each residue on the central residue. We extract ${RACF\left(l\right)}^{i}\;and\;\rho_{l}^{i}\left(k\right)\;\left(l=1,2,\ldots ,20\right)$ to describe the effect of the whole formed by a certain number of residues on the central residue and the effect of each residue in the whole on the central residue. We also take the five geometric values of residue as features to describe the central residue. So we can use 5×(20+20+20)+5 = 305 features to describe each residue.

In summary, for each amino acid (residue), we can extract 155+305 = 460 features, and combine these 460 features to form a feature vector *U*. Therefore, we use a 920 dimensional feature vector to represent a residue pair. Taking the residue pairs generated by residues on A chain and B chain of the 1DD3 tetramer protein complex as an example. We use $U_{j}^{A}$ to represent the feature vector of the residue *j* on A chain and use $U_{k}^{B}$ to represent the feature vector of the residue *k* on B chain. Then we use $U1=\left(U_{j}^{A},U_{k}^{B}\right)$ to represent the residue pair generated by residue *j* on A chain and residue *k* on B chain. We use Y to represent the sample label (Y = 1 indicates that the residue pair is an interface residue pair, Y = 0 indicates that the residue pair is a non-interface residue pair).

#### 2.3.2 SVM ensemble method

Support Vector Machine (SVM) has been widely used in the study of protein-protein interactions, and has achieved good results. In this paper, we also use SVM to predict the tetramer protein complex interface residue pairs. Compared with non-interface residue pairs, the number of interface residue pairs is very small in tetramer protein complex. Therefore, the positive and negative classes are very imbalanced in the date set (positive class: interface residue pair, negative class: non-interface residue pair). We take under-sampling method to deal with the class imbalance problem and use ensemble method to reduce the information loss caused by under-sampling.

**FIGURE 4:**
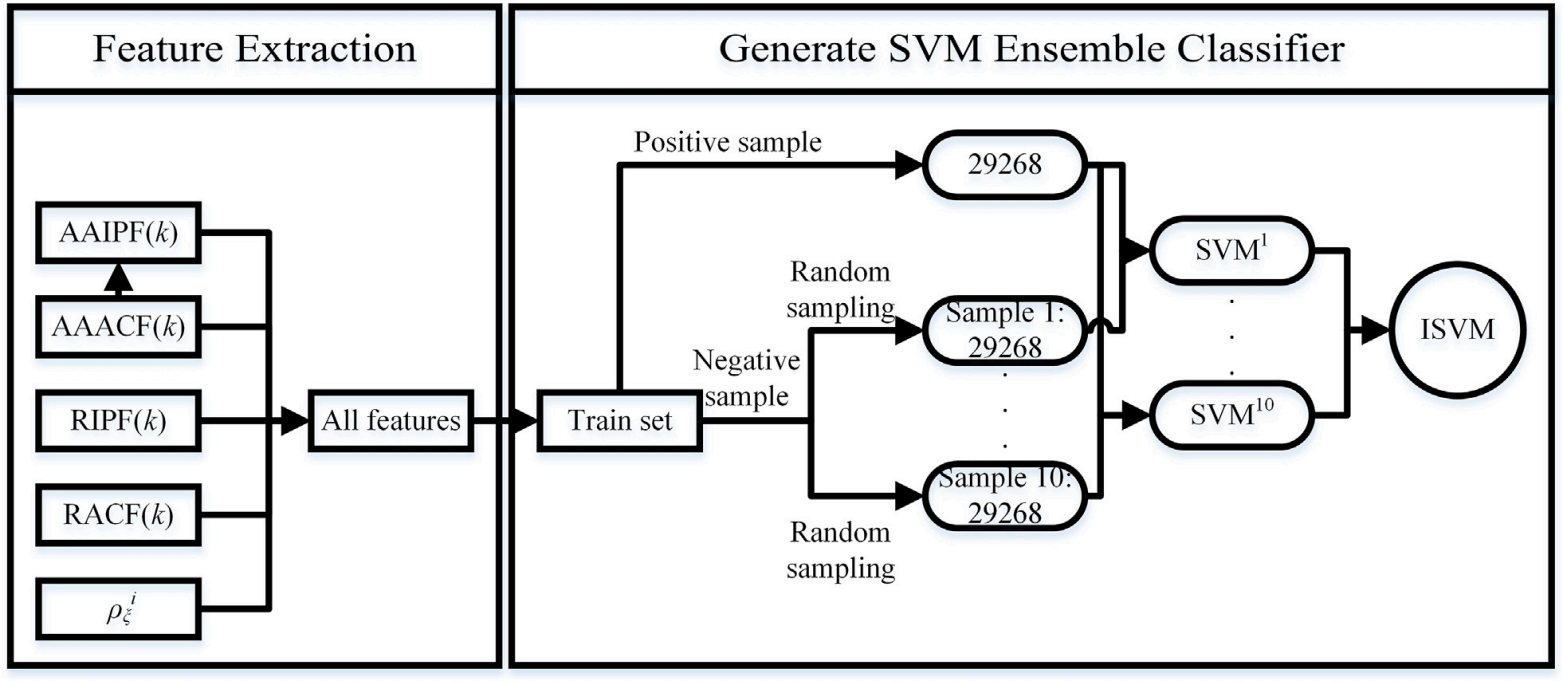
Flow chart of the SVM Ensemble Method.

We propose SVM ensemble method to predict the tetramer protein complex interface residue pairs. Our method can be divided into two parts: Feature Extraction and Generate SVM Ensemble Classifier (see Figure 4). The feature extraction is shown in Section 2.3.1. The process of generate SVM ensemble classifier is as follows:

The total number of positive samples in training set is 29,268. We randomly sample 10 times from all negative samples to generate 10 subsets. And we set the number of negative samples per random sampling to 29268. Then we combine each subset of negative samples with all positive samples to generate a balanced sample set. We obtain 10 balanced sample sets. By training the SVM model with each balanced sample set, 10 independent SVM models can be obtained. Finally, we use an integration strategy to fuse 10 independent SVM models to generate a SVM ensemble classifier ISVM, see formula 24. In the SVM model, SVM type is C-classification, SVM kernel function is radial basis function.

$$ISVM\left(x\right)=\sum_{\psi =1}^{10}{SVM}^{\psi }\left(x\right)$$ (24)

Where ${SVM}^{\psi }$ represents the SVM predictor trained with the ψ -th balanced sample set. *x* represents a residue pair. ${SVM}^{\psi }\left(x\right)$ represents the probability that the ψ -th individual SVM model predicts that the residue pair *x* is an interface residue pair.

## 3 Results

### 3.1 Predictions of the interaction between chains of the tetramer protein complex

#### 3.1.1 Evaluation criteria

We use 7 common evaluation indicators (recall, specificity, precision, F1 score, Matthews Correlation Coefficient (MCC), accuracy and AUC) to evaluate the predictions. Their definitions as follows:

$$Recall=\frac{TP}{TP+FN}$$ (25)

$$Specificity=\frac{TN}{TN+FP}$$ (26)

$$Precision=\frac{TP}{TP+FP}$$ (27)

$$F1=\frac{2TP}{2TP+FP+FN}$$ (28)

$$MCC=\frac{TP\times TN-FP\times FN}{\sqrt{\left(TP+FP\right)\left(TP+FN\right)\left(TN+FP\right)\left(TN+FN\right)}}$$ (29)

$$Accuracy=\frac{TP+TN}{TP+TN+FP+FN}$$ (30)

Where, *TP* indicates the number of positive samples predicted by the model to be positive class. *FN* indicates the number of positive samples predicted by the model to be negative class. *FP* indicates the number of negative samples predicted by the model to be positive class. *TN* indicates the number of negative samples predicted by the model to be negative class.

We also define a new evaluation indicator ||*PT*||_1_ to evaluate our predictions. ||*PT*||_1_ represents the L1 norm of the vector *PT*, which means the sum of the number of interactions and non-interactions correctly predicted in a tetramer protein complex. *PT*=(a, b), where a represents the number of correctly predicted interactions in a tetramer protein complex, and b represents the number of correctly predicted non-interactions in a tetramer protein complex.

#### 3.1.2 Results

We randomly divide 111 tetramer protein complexes into the training set, verification set and testing set, of which the number of tetramer protein complexes in training set is 63, the number of tetramer protein complexes in validation set is 20, and the number of tetramer protein complexes in testing set is 28 (see Supplementary Table S1). The number of positive and negative samples in each data set is shown in Table 1.

**TABLE 1:** Information of positive and negative samples in each data set.

	Positive sample number	Negative sample number
Training set	310	68
Validation set	105	15
Testing set	149	19

Input feature maps of training set and testing set into the CNN model to train the hyper parameters and verify the accuracy of the model. The hyper parameters finally selected are as follows: the learning rate is 0.000801, the number of convolution kernels is 2, and the number of epoch is 20. Under the above hyper parameters, the results of CNN model on validation set and testing set is shown in Table 2.

**TABLE 2:** Predictions of CNN Model on validation set and testing set.

	Recall	Specificity	Precision	F1	MCC	Accuracy	AUC
Validation set	0.9369	0.3333	0.9455	0.9412	0.2576	0.8917	0.7608
Testing set	0.9329	0.2105	0.9026	0.9175	0.1643	0.8512	0.6263

It can be seen from Table 2 that the recall of validation set and testing set is 0.9369 and 0.9329 respectively, which indicates that our CNN model is relatively accurate in predicting the interaction between two chains of the tetramer protein complex. The specificity of validation set and testing set is 0.3333 and 0.2105 respectively. The precision of the verification set and testing set is 0.9455 and 0.9026 respectively. The MCC of the verification set and testing set is 0.2576 and 0.1643 respectively. As the data of non-interactions between two chains in tetramer protein complexes is too sparse, the specificity and MCC values in validation set and testing set are relatively low. The F1 value of the verification set and testing set is 0.9412 and 0.9174 respectively. The AUC value of validation set and testing set is 0.7608 and 0.6263 respectively. Through the analysis of the above results, it shows that our CNN model can distinguish the positive and negative sample, that is, the method can be used to predict the interaction between chains of the tetramer protein complex.

The specific prediction of each tetramer protein complex in testing set is shown in Table 3. It can be seen that 15 tetramer protein complexes in testing set only have interaction between chains. The CNN model can correctly predict 9 of them, with an accuracy of 60%. The number of interactions between two chains in testing set is 149. The CNN model can correctly predict 139 of them, with an accuracy of 93.29%. For the 6 samples formed by each tetramer protein complex, at least 5 samples can be correctly predicted by CNN model, with an accuracy of 82.14%. The results also show that the CNN model can distinguish the positive and negative samples in 1DD3, 1P27, 1ZXJ and 3SQO tetramer protein complexes.

**TABLE 3:** Prediction of each tetramer protein complex in testing set.

PDB ID	*a* ^a^	*b* ^a^	||*PT*||_1_	relationship^b^
1DD3	2	1	3	2
1F5Z	4	0	4	2
1J2W	5	0	5	1
1NSW	5	0	5	2
1P27	3	1	4	2
1QVC	6	0	6	1
1QYN	6	0	6	1
1REW	5	0	5	2
1SWF	5	0	5	1
1UDD	5	0	5	1
1UFQ	6	0	6	1
1WYT	6	0	6	1
1ZXJ	3	1	4	2
2A2U	6	0	6	1
2EPI	6	0	6	1
2OZK	5	0	5	2
2Z8U	5	0	5	2
2ZIH	5	0	5	2
2ZME	5	0	5	2
2ZYZ	5	0	5	2
3HM0	6	0	6	1
3IBF	6	0	6	1
3ITY	6	0	6	1
3KYH	5	0	5	2
3SQO	4	1	5	2
3STB	5	0	5	1
3V15	5	0	5	1
3VH5	4	0	4	1

^a^ There are maybe two kinds of relationship that is interaction and non-interaction between any two chains in each tetramer protein complex. In Table 3, a represents the number of interactions that are correctly predicted in each tetramer protein complex, and b represents the number of non-interactions that are correctly predicted in each tetramer protein complex.

^b^ The number 1 in b column indicates that there is only interaction between chains in the tetramer protein complex. The number 2 in b column indicates that there are both interaction and non-interaction relationships between chains in the tetramer protein complex.

### 3.2 Predictions of the tetramer protein complex interface residue pairs

#### 3.2.1 Evaluation criteria

The output value of SVM ensemble method is between 0 and 1, which indicates the possibility that the residue pair is an interface residue pair. The predicted values are arranged in descending order. We take the *t* predictions with the highest probability as the predicted *t* interface residue pairs.

In addition to recall, specificity, precision, F1 score, MCC and AUC, these six commonly indicators. In this part, we also define three new indicators to evaluate the performance of SVM ensemble method. Before introducing these three new indexes, we define a six dimensional vector ${NPIRP}^{4}\left(t\right)={\left(n_{1},n_{2},n_{3},n_{4},n_{5},n_{6}\right)}_{t}$ , Where $n_{z}$ represents the number of positive interface residue pairs in the top *t* predictions of the *Z*th possible protein-protein interaction interface in the tetramer protein complex. Based on this six dimensional vector, we give definitions of three indicators, as follows

The first index is ${\left\|{NPIRP}^{4}\left(t\right)\right\|}_{0}$ , representing the L0 norm of the vector ${NPIRP}^{4}\left(t\right)$ , which is consistent with the meaning of the vector L0 norm in mathematics. The biological meaning of ${\left\|{NPIRP}^{4}\left(t\right)\right\|}_{0}$ is the number of correctly predicted protein-protein interaction interfaces in each tetramer protein complex. If there is at least one positive interface residue pair in the top *t* predictions, we consider that the protein-protein interaction interface is correctly predicted.

The second index is ${\left\|{NPIRP}^{4}\left(t\right)\right\|}_{1}$ (see formula 31), representing the L1 norm of the vector ${NPIRP}^{4}\left(t\right)$ , which is consistent with the meaning of the vector L1 norm in mathematics. The biological meaning of ${\left\|{NPIRP}^{4}\left(t\right)\right\|}_{1}$ is the number of correctly predicted interface residue pairs in the top *t* predictions at a tetramer protein complex.

$${\left\|{NPIRP}^{4}\left(t\right)\right\|}_{1}=\overset{6}{\underset{z=1}{\sum }}n_{z}$$ (31)

The third index is ${Accuracy}^{4}\left(t\right)$ , see formula 32.

$${Accuracy}^{4}\left(t\right)=\frac{NCTP\left(t\right)}{NTP}\times 100\%\;$$ (32)

Where $NCTP\left(t\right)$ represents the Number of Correctly predicted Tetramer Protein complexes. In the top *t* predictions, we consider that the tetramer protein complex is correctly predicted, when there are *z* protein–protein interaction interfaces that each interface has at least one positive interface residue pair. *NTP* represents the Number of Tetramer Protein complexes containing at least *z* native protein-protein interaction interfaces in the data set.

#### 3.2.2 Results

We randomly divide 111 tetramer protein complexes into training set and testing set according to the ratio of about 3:1. Training set contains 83 tetramer protein complexes and testing set contains 28 tetramer protein complexes. The tetramer protein complexes PDB ID of each set is shown in Supplementary Table S1. The specific number of positive and negative samples in each set is shown in Table 4.

**TABLE 4:** Sample number information of training set and testing set.

	Positive sample	Negative sample	Total sample	Positive sample proportion
Training set	29268	22525471	22554739	0.001298
Testing set	10862	7040217	7051079	0.00154

Firstly, the feature vector of each residue pair is calculated and the specific process see Section 2.3.1. Secondly, we use the samples generated by training set to train model. Then, the samples generated by testing set are input into the training model. Finally, we obtain the score of each residue pair in testing set.

Table 5 shows two evaluation indexes ${\left\|{NPIRP}^{4}\left(t\right)\right\|}_{0}$ and ${\left\|{NPIRP}^{4}\left(t\right)\right\|}_{1}$ of testing set. From Table 5 we get the following conclusions: In the top 10 predictions, when at least one protein-protein interaction interface in a tetramer protein complex is correctly predicted, a total of 23 tetramer protein complexes are correctly predicted, when at least two protein-protein interaction interfaces in each tetramer protein complex is correctly predicted, a total of 20 tetramer protein complexes are correctly predicted. In the top 30 predictions, when at least three protein-protein interaction interfaces in each tetramer protein complex are correctly predicted, a total of 20 tetramer protein complexes are correctly predicted, when at least four protein-protein interaction interfaces in each tetramer protein complex are correctly predicted, a total of 16 tetramer protein complexes are correctly predicted.

**TABLE 5:** Two evaluation indexes of testing set in the top *t* predictions.

PDB	*t* = 10		*t* = 15		*t* = 20		*t* = 30	
ID	||*NPIRP* ^4^||_0_	||*NPIRP* ^4^||_1_	||*NPIRP* ^4^||_0_	||*NPIRP* ^4^||_1_	||*NPIRP* ^4^||_0_	||*NPIRP* ^4^||_1_	||*NPIRP* ^4^||_0_	||*NPIRP* ^4^||_1_
1DD3	2	3	2	6	4	8	4	14
1F5Z	0	0	2	2	4	4	4	9
1J2W	2	2	2	4	3	6	4	11
1NSW	4	13	4	14	5	22	5	31
1P27	1	2	2	6	2	8	2	12
1QVC	0	0	3	3	3	3	6	12
1QYN	4	8	5	12	6	16	6	21
1REW	2	4	2	5	2	9	2	16
1SWF	4	15	5	18	6	22	6	26
1UDD	2	2	2	2	2	4	3	7
1UFQ	2	5	4	7	4	9	4	13
1WYT	1	1	1	2	2	3	2	3
1ZXJ	0	0	0	0	0	0	0	0
2A2U	2	2	2	2	2	2	4	4
2EPI	6	9	6	12	6	14	6	19
2OZK	2	5	2	5	2	6	2	6
2Z8U	0	0	0	0	2	2	2	2
2ZIH	2	4	2	4	3	5	3	5
2ZME	3	3	3	3	3	7	4	11
2ZYZ	2	2	3	5	3	6	4	11
3HM0	4	8	6	13	6	14	6	17
3IBF	3	3	3	4	3	5	3	7
3ITY	4	4	4	5	4	5	4	5
3KYH	0	0	0	0	0	0	1	1
3SQO	1	1	1	1	1	1	2	2
3STB	4	13	4	14	4	15	5	22
3V15	3	7	3	9	3	12	3	17
3VH5	5	13	5	14	5	15	5	17

In the top 10 predictions, the prediction of 2EPI tetramer protein complex is the best. Six protein-protein interaction interfaces are correctly predicted, and a total of 9 positive interface residue pairs are given. The prediction of 3VH5 tetramer protein complex follows closely. Five protein-protein interaction interfaces are correctly predicted, and a total of 13 positive interface residue pairs are given. On 1SWF tetramer protein complex, four protein-protein interaction interfaces are correctly predicted, and a total of 15 positive interface residue pairs are given. On 1NSW and 3STB tetramer protein complexes, four protein-protein interaction interfaces are correctly predicted, and a total of 13 positive interface residue pairs are given.

We calculate the index ${Accuracy}^{4}\left(t\right)$ according to the ${\left\|{NPIRP}^{4}\left(t\right)\right\|}_{0}$ columns in Table 5(see Table 6). As can be seen from Table 6, in the top 10 predictions, when at least one protein-protein interaction interface is correctly predicted for each tetramer protein complex, the ${Accuracy}^{4}\left(t\right)$ of SVM ensemble method is 82.14%, that is about 4/5 of tetramer protein complexes in testing set can be correctly predicted. In the top 20 predictions, when at least two protein–protein interaction interfaces are correctly predicted for each tetramer protein complex, the ${Accuracy}^{4}\left(t\right)$ of SVM ensemble method is 89.29%. In the top 30 predictions, when at least four protein–protein interaction interfaces are correctly predicted for each tetramer protein complex, the ${Accuracy}^{4}\left(t\right)$ of SVM ensemble method is 61.43%, that is about 3/5 of the tetramer protein complexes in testing set could be correctly predicted.

**TABLE 6:** ${Accuracy}^{4}\left(t\right)$ of testing set prediction.

*z*	*t* = 10 (%)	*t* = 15 (%)	*t* = 20 (%)	*t* = 30 (%)
*z* = 1	82.14	89.29	92.86	96.43
*z* = 2	71.43	82.14	89.29	92.86
*z* = 3	39.29	50.00	64.29	71.43
*z* = 4	30.77	34.62	42.31	61.54
*z* = 5	8.33	20.83	25.00	33.33
*z* = 6	6.67	13.33	26.67	33.33

When we give the top 50 predictions and all native protein-protein interaction interfaces on each tetramer protein complex are required to be correctly predicted, SVM ensemble method can correctly predict 9 tetramer protein complexes. The predictions of these 9 tetramer protein complexes are shown in Table 7. The prediction of 1NSE tetramer protein complex is the best. A total of 53 positive interface residue pairs are given on 5 native protein-protein interaction interfaces, with an average of 10.6 positive interface residue pairs per protein-protein interaction interface. On 1QYN, 1SWF and 3MH0 tetramer protein complexes, SVM ensemble method gives at least 30 positive interface residue pairs, with an average of 5 positive interface residue pairs per protein-protein interaction interface.

**TABLE 7:** Predictions of 9 tetramer protein complexes in testing set.

PDB ID	Native interface number^a^	Positive interface residue pair^b^
1F5Z	4	10
1J2W	6	20
1NSW	5	53
1QVC	6	22
1QYN	6	32
1SWF	6	34
1UDD	6	21
2EPI	6	24
3HM0	6	30

^a^ Represents the number of native protein-protein interaction interfaces in each tetramer protein complex, and all these protein-protein interaction interfaces are correctly predicted by SVM ensemble method.

^b^ Represents the number of positive interface residue pairs correctly predicted by SVM ensemble method on each tetramer protein complex.

In the top 200 predictions, the recall, precision, specificity, F1 and MCC of SVM ensemble method are 0.255, 0.052, 0.988, 0.081 and 0.102 respectively. In fact, if 200 residue pairs per protein-protein interaction interface are taken as interface residue pairs, a total of 29800 residue pairs are extracted as interface residue pairs in testing set. According to the proportion of interface residue pairs in the total residue pairs of testing set, there should be 45.98 interface residue pairs in the 29800 residue pairs, and the precision is 0.00154, the precision of SVM ensemble method is much higher than this value. Compared with non-interface residue pairs, interface residue pairs are too sparse, so the precision and F1 value of SVM ensemble method are not high.

In reference (Sun and Gong, 2020), Sun et al. predicted the tetramer protein complex interface residue pairs based on LSTM network with a graph. We compare the performance of our method with Sun et al. method (using optimal super parameters). In the top 10 predictions, when at least one protein-protein interaction interface is correctly predicted, the accuracy of our method is 82.14% and Sun et al. method is 83.33%, when at least two protein-protein interaction interfaces are correctly predicted, the accuracy of our method is the same as that of Sun et al., when at least three protein-protein interaction interfaces are correctly predicted, the accuracy of our method is 30.29% and Sun et al. method is 25.84%. In the top 20 predictions, when at least one protein-protein interaction interface is correctly predicted, the accuracy of our method and Sun et al. method is same, which is 92.86%, when at least two protein-protein interaction interfaces are correctly predicted, the accuracy of our method is 89.29% and Sun et al. method is 85.71%, when at least three protein-protein interaction interfaces are correctly predicted, the accuracy of our method is 64.29% and Sun et al. method is 61.54%. It can be seen that the predictions of our method are better than those of Sun et al. on the whole.

## 4 Discussion

In this paper, we have done two parts of work to predict the tetramer protein complex interaction. In the first part, we defined the position change sequence and geometric feature change sequences of the same type of amino acid. Based on these sequences, we proposed a 20 × 24 feature map to represent a sample generated by two chains in a tetramer protein complex and constructed a CNN model to predict the interaction between chains of the tetramer protein complex. In the second part, we considered the influence of surrounding amino acids (residues) on the central amino acid (the central residue) when extracting features. We defined Amino Acid *k*-Average Cumulation Factor, together with Amino Acid *k*-Interval Product Factor to extract features based on protein sequence. We also defined the Residue *k*-Interval Product Factor, Residue *k*-Average Cumulation Factor and weight factor to extract features based on protein three-dimensional structure. Finally, we proposed a SVM ensemble method based on under-sampling and ensemble method to predict the tetramer protein complex interface residue pairs. The prediction shows that our method is feasible for the prediction of tetramer protein complex interface residue pairs. Compared with previous studies, which only studied tetramer protein complex interface residue pairs, we also studied the interaction between chains of the tetramer protein complex, which provides a new perspective for the future study of multibody protein interactions. However, there are also the following points that need to be further improved. The first point is the study of the interaction between chains of the tetramer protein complex, whose accuracy still needs to be further improved. The second point, when all native protein-protein interaction interfaces of each tetramer protein complex can be correctly predicted, our accuracy also needs to be further improved. In the future, we also hope that our predictions can be used in docking processes to predict the multibody protein complex three-dimensional structure.

## Data Availability

The original contributions presented in the study are included in the article/Supplementary Material, further inquiries can be directed to the corresponding authors.
