# Supplementary material for: Prediction of the tetramer protein complex interaction based on CNN and SVM
Source: Front Genet. 2023 Jan 26;14:1076904. doi: 10.3389/fgene.2023.1076904 (PMC9909274; doi:10.3389/fgene.2023.1076904)
Supplement: Supplementary file 1 [file DataSheet1.PDF]

Supplementary Material

Prediction of the tetramer protein complex interaction based on CNN and SVM

Yanfen Lyu 1, Chunxia Wang3*, Xinqi Gong 4, 5*

*** Correspondence:** Chunxia Wang: wangchunxia@hebeu.edu.cn;

Xinqi Gong: xinqigong@ruc.edu.cn

1 Supplementary Tables

Table S1 PDB ID of tetramer protein complex

Data set name	PDB ID							
Training set	1A4Y	1AGQ	1B35	1B79	1BDF	1BML	1BV4	1C4P
	1E65	1FS1	1FS2	1FX3	1GPQ	1J1J	1JL2	1JQJ
	1KAM	1L3A	1LBI	1M1L	1N5B	1OFT	1PV1	1Q15
	1Q5V	1QSO	1QUQ	1SWH	1SD0	1U9Y	1UDR	1UFB
	1VL2	1WWH	1Y14	1YIF	2ACI	2AHD	2AN1	2D3E
	2D7E	2E3D	2E6E	2EP5	2H3N	2H8N	2IJ0	2JBR
	2NNW	2NQ2	2NQO	2NRC	2OGK	2OKA	2PBY	2QIY
	2QW6	2R90	2X3W	3AGQ	3BF0	3CO2	3CUQ	
Validation set	1AZZ	1FTR	1TJV	1U4F	2GAC	2XHZ	2YWB	3B8F
	3DMP	3ESI	3F6Z	3G33	3G7K	3GXV	3IB6	3IWV
	3LHF	3QTL	3RD4	3TUO				
Testing set	1DD3	1F5Z	1J2W	1NSW	1P27	1QVC	1QYN	1REW
	1SWF	1UDD	1UFQ	1WYT	1ZXJ	2A2U	2EPI	2OZK
	2Z8U	2ZIH	2ZME	2ZYZ	3HM0	3IBF	3ITY	3KYH
	3SQO	3STB	3V15	3VH5				

Table S2. Five physicochemical properties for 20 amino acids.

Amino acid	Φ^1	Φ^2	Φ^3	Φ^4	Φ^5
A	0.62	0.046	8.1	−1.302	1.57
C	0.29	0.128	5.5	0.465	−1.02
D	−0.9	0.105	13	0.302	−0.259
E	−0.74	0.151	12.3	−1.453	0.113
F	1.19	0.29	5.2	−0.59	−0.397
G	0.48	0	9	1.652	1.045
H	−0.4	0.23	10.4	−0.417	−1.474
I	1.38	0.186	5.2	−0.547	0.393
K	−1.5	0.219	11.3	−0.561	−0.277
L	1.06	0.186	4.9	−0.987	1.266
M	0.64	0.221	5.7	−1.524	−1.005
N	−0.78	0.134	11.6	0.828	−0.169
P	0.12	0.131	8	2.081	0.421
Q	−0.85	0.18	10.5	−0.179	−0.503
R	−2.53	0.291	10.5	−0.055	0.44
S	−0.18	0.062	9.2	1.399	0.67
T	−0.05	0.108	8	0.326	0.908
V	1.08	0.14	5.9	−0.279	1.242
W	0.81	0.409	5.4	0.009	−2.128
Y	0.26	0.298	6.2	0.83	−0.838

[1]We use Φ^1, Φ^2, Φ^3, Φ^4 and Φ^5 to represent the hydrophobicity, polarizability, polarity, secondary structure, codon diversity of 20 amino acids.
